# Investigating the correspondence between driver head position and glance location

**DOI:** 10.7717/peerj-cs.146

**Published:** 2018-02-19

**Authors:** Joonbum Lee, Mauricio Muñoz, Lex Fridman, Trent Victor, Bryan Reimer, Bruce Mehler

**Affiliations:** 1 AgeLab and New England University Transportation Center, Massachusetts Institute of Technology, Cambridge, MA, United States of America; 2 Technical University of Munich, Munich, Germany; 3 University of Augsburg, Augsburg, Germany; 4 SAFER Vehicle and Traffic Safety Center, Chalmers, Göteborg, Sweden

**Keywords:** Head movements, Glance classification, Head-glance correspondence, Driver distraction

## Abstract

The relationship between a driver’s glance orientation and corresponding head rotation is highly complex due to its nonlinear dependence on the individual, task, and driving context. This paper presents expanded analytic detail and findings from an effort that explored the ability of head pose to serve as an estimator for driver gaze by connecting head rotation data with manually coded gaze region data using both a statistical analysis approach and a predictive (i.e., machine learning) approach. For the latter, classification accuracy increased as visual angles between two glance locations increased. In other words, the greater the shift in gaze, the higher the accuracy of classification. This is an intuitive but important concept that we make explicit through our analysis. The highest accuracy achieved was 83% using the method of Hidden Markov Models (HMM) for the binary gaze classification problem of (a) glances to the forward roadway versus (b) glances to the center stack. Results suggest that although there are individual differences in head-glance correspondence while driving, classifier models based on head-rotation data may be robust to these differences and therefore can serve as reasonable estimators for glance location. The results suggest that driver head pose can be used as a surrogate for eye gaze in several key conditions including the identification of high-eccentricity glances. Inexpensive driver head pose tracking may be a key element in detection systems developed to mitigate driver distraction and inattention.

## Introduction

Eye movements have long been studied in the context of driver behavior, attention management, and task related visual demand assessment (e.g., Wierwille, 1993). As driver distraction has become identified as one of the leading causes of vehicle crashes (National Highway Traffic Safety Administration, 2015; Statistic Brain, 2017), eye-tracking systems have been employed in numerous studies in the field of driving safety and driver visual attention allocation (e.g., Wang et al., 2014). Evaluating objective data on glance behavior, such as glance location (e.g., glance to the road, glance away from the road, etc.) and duration (e.g., mean of single glance duration, total glance time to a specific location, etc.) has been seen as key to understanding driver interaction with in-vehicle devices and to estimate potential crash risks. For example, several studies show that crash and near-crash risk increased as the duration of off-road glances increased (e.g., Liang, Lee & Yekhshatyan, 2012; Victor et al., 2015). However, traditional technologies for automated eye-tracking have been susceptible to data quality issues (Ahlstrom et al., 2012; Sodhi, Reimer & Llamazares, 2002) and difficult to reliably use in production systems, especially for on-road experiments and naturalistic driving studies. For these reasons, research on the correspondence between eye and head movement (which is relatively more robust to track in the presence of occlusion and movement artifact) have been conducted, and results suggest that head pose data may be useful as a surrogate for eye-glance data (e.g., Talamonti et al., 2013; Talamonti, Kochhar & Tijerina, 2014; Tawari, Chen & Trivedi, 2014; Vicente et al., 2015), although there may be issues as well. Talamonti and colleagues (2013) found a low likelihood (65% or less) of head turns when glancing to the instrument panel and rearview mirror, and high likelihood (93% or more) when glancing to the left mirror, center console, and center stack. Also, Talamonti, Kochhar & Tijerina (2014) suggested that driver-specific thresholds need to be set in order to meaningfully use head yaw data as a glance predictor. These studies utilized a fixed-base driving simulator to collect data and applied a simple classifier to understand the relationship between head turns and glance locations. Tawari, Chen & Trivedi (2014) and Vicente et al. (2015) applied more advanced approaches, extracting facial features and landmarks to estimate gaze regions while driving a real car, but they did not focus on driver distraction aspects (e.g., completing secondary tasks while driving), which have critical safety implications.

The present paper presents expanded analytic detail and findings from an effort that was developed to further explore whether head-rotation data can be used as a surrogate for eye-glance behaviors in an on-road environment where eye-tracking is more challenging compared to laboratory experiments (Muñoz et al., 2015). This analysis took advantage of glance and head rotation data drawn from a study conducted by the Virginia Tech Transportation Institute (Transportation Research Board, 2013); the glance data were manually coded for glance region and temporal points where glance orientation transition from one location to another by two independent coders (and a senior research associated mediated if disagreement occurred between two coders), and head rotation data were estimated from manually extracted facial landmarks (details are described in the ‘Method’ section). This study utilized the data to: (a) begin developing a deeper understanding of how drivers’ rotate their heads, (b) generate input features for classifiers that predicted glance allocations, and (c) investigate individual differences in head-glance correspondence. Based on the literature noted above, it was expected that head-rotation data could be used to predict some, but not all, glances away from the road. In the field of driver distraction evaluation, glances away from the road and glances to task-related areas such as displays are particularly important to measure (Driver Focus-Telematics Working Group, 2006; National Highway Traffic Safety Administration, 2015). Therefore, we tested whether head rotation can predict glances to the forward road, to the vehicle’s center stack (e.g., climate controls, infotainment display), and to other key locations in the vehicle (e.g., mirrors, see Li & Busso, 2016). Subsequent efforts then evaluated the degree to which machine learning algorithms could predict glances to closer and farther regions of the vehicle interface and to evaluate the degree to which individual differences influence behavior.

The main objective of this study is to investigate the use of head pose data to predict glance location with on-road driving data. To achieve this, we analyze the data using principal component analysis (PCA) and machine learning techniques by considering several factors that may affect model performance and interpretation. Classification performance is a direct result of three principal factors: (a) the quantity and “shape” (e.g., uneven class membership, skewness, etc.) of the data, (b) the modeling methodology utilized, and (c) the descriptive potential (i.e., signal power) of the selected features. We consider results based on the original “skewed” dataset, which is characterized by a heavily uneven distribution of samples for each glance type (95% of all glances were forward glances), as well as a subset of the original dataset with an equal amount of glance samples for each type of glance. Furthermore, in terms of model selection, there is a wide range of classifiers that could be selected from. As a secondary assessment of the viability of different classification approaches, four classifiers were examined to cover a wide range of data interpretation paradigms. These steps allow us to reasonably begin to isolate the descriptive potential of the head pose signal and a set of classification approaches that appear most promising for future endeavors targeting larger more sophisticated datasets or systems for real-time state assessment. Subsequent sections describe details of the data and model development.

## Methods

As previously noted, this study is a secondary analysis of a subset of data collected by the Virginia Tech Transportation Institute (VTTI) in support of the Strategic Highway Research Program 2 (SHRP 2) naturalistic driving study (Transportation Research Board, 2013). The data were provided to the MIT AgeLab under an IRB approved data sharing agreement. A number of the following details concerning the source dataset are drawn from a technical report (J Sudweeks, A Sarkar & A Plummer, 2014, unpublished data) prepared by VTTI for the SHRP 2 program. Participants were initially recruited to ensure that the dataset represented a wide array of facial geometry. Participants who met the study’s eligibility criteria were assigned to participate in either static trials (e.g., data collected while not driving) or dynamic trials (e.g., data collected while driving). A total of 44 participants were available (22 participants for static trials and 22 participants for dynamic trials). The sample spans four age groups (18–35, 36–50, 51–65, and over 66, with a majority of cases falling in the first two groups) and consisted of 30 males and 14 females. Data were collected in a 2001 Saab 9-3 instrumented with a data acquisition system to collect a number of metrics, including digital video of the driver’s face. The video was recorded by a camera mounted below the rearview mirror. A previous brief report from our group (Muñoz et al., 2015) showed a number of differences in the distributions of head rotations associated with glances to the road and center cluster between the static and dynamic samples. Consequently, the data from the 22 participants from the dynamic trials make up the focus of this analysis since actual on-road behavior is our primary interest.

### Test trials

The dynamic trials were conducted on a predefined route around Blacksburg, Virginia. This route was approximately 15 miles in length and consisted of various road types (e.g., two lane road, residential, rural, and divided highway). During the driving session, which usually lasted between 60 to 90 min, participants were instructed to perform five basic tasks, each of which was performed once: (a) report current vehicle speed, (b) indicate if any vehicles are immediately adjacent to the test vehicle, (c) turn the radio on and then off, (d) locate the cell phone in the center console, and (e) complete a brief simulated cell phone conversation. Participants were accompanied by an in-vehicle experimenter who instructed participants to conduct each of the tasks at a safe time at roughly the same location on the route.

### Data reduction

**Figure 1:**
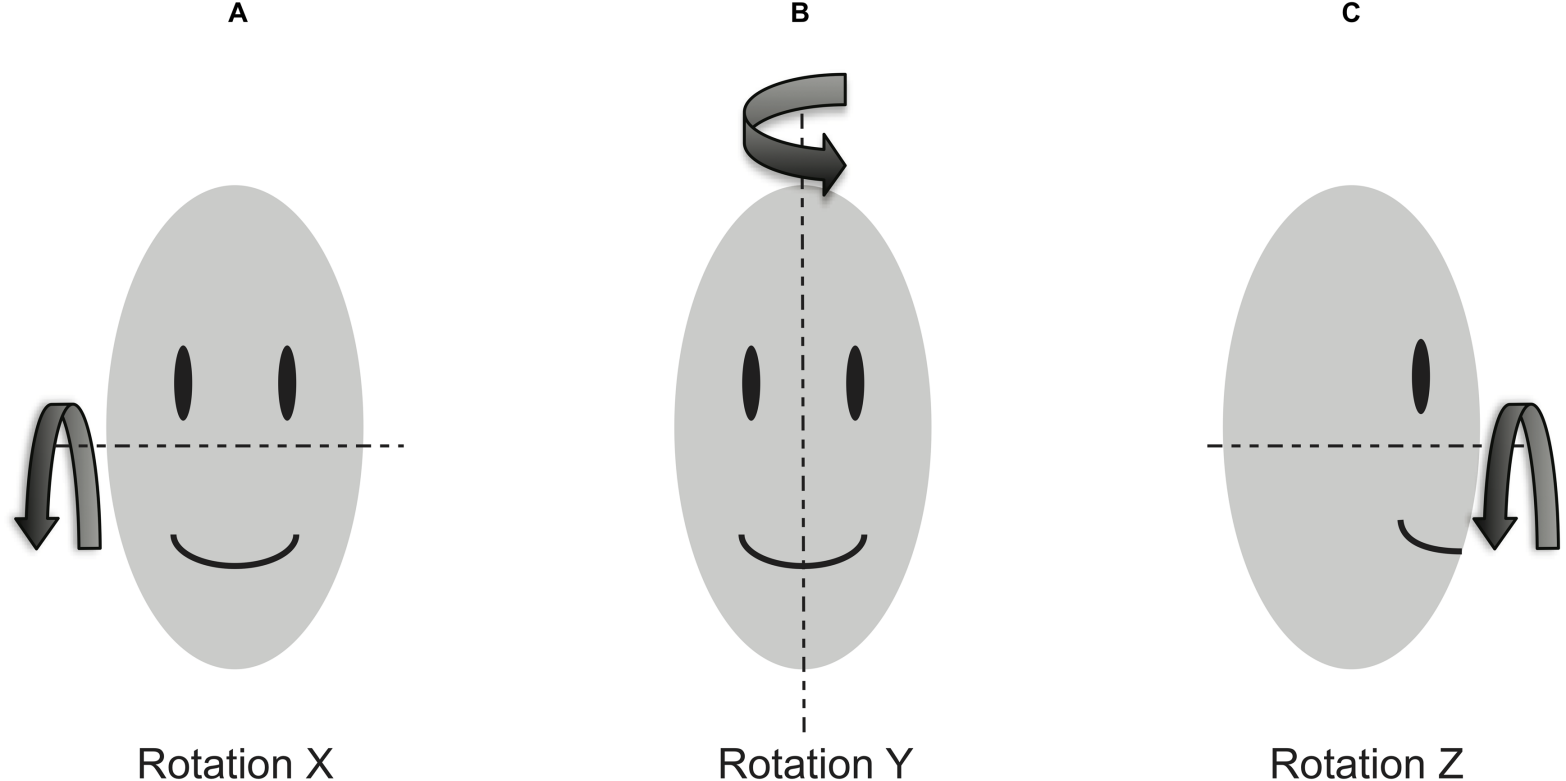
Head rotation coordinate system. (A) Rotation *X*: Pitch, (B) Rotation *Y*: Yaw, and (C) Rotation *Z*: Roll.

Video of each task/glance was recorded at 15 frames per second and decomposed into frames for analysis. Each video frame was annotated by two independent analysts who labeled seven predefined facial landmarks: (a) outer corner of the participant’s right eye, (b) inner corner of the participant’s right eye, (c) outer corner of the participant’s left eye, (d) inner corner of the participant’s left eye, (e) the tip of the participant’s nose, (f) the right corner of the participant’s mouth, and (g) the left corner of the participant’s mouth. Two analysts’ *x* and *y* pixel coordinates for each landmark were averaged, and if the average frame pixel correction exceeded 3.5 pixels, the frame was considered as a significant disagreement between two analysts, and was excluded from the rotation estimate dataset. If either analyst could not make a reliable annotation, the landmark was marked as “missing,” and the frame was excluded from the rotation estimate dataset. For each video frame, geometric methods (e.g., Murphy-Chutorian & Trivedi, 2009; Gee & Cipolla, 1994; Horprasert, Yacoob & Davis, 1996), which utilize feature locations (e.g., eyes, mouth, and nose tip), configurations of the facial features, basic ratios between feature locations (e.g., ratio between binocular width and vertical distance from lit to midpoint between eyes), etc. were used for head rotation estimation. The head pose data consisted of three rotation estimates (i.e.,  *X*, *Y* and *Z* rotation). Figure 1 shows a rotation coordinate system.

**Figure 2:**
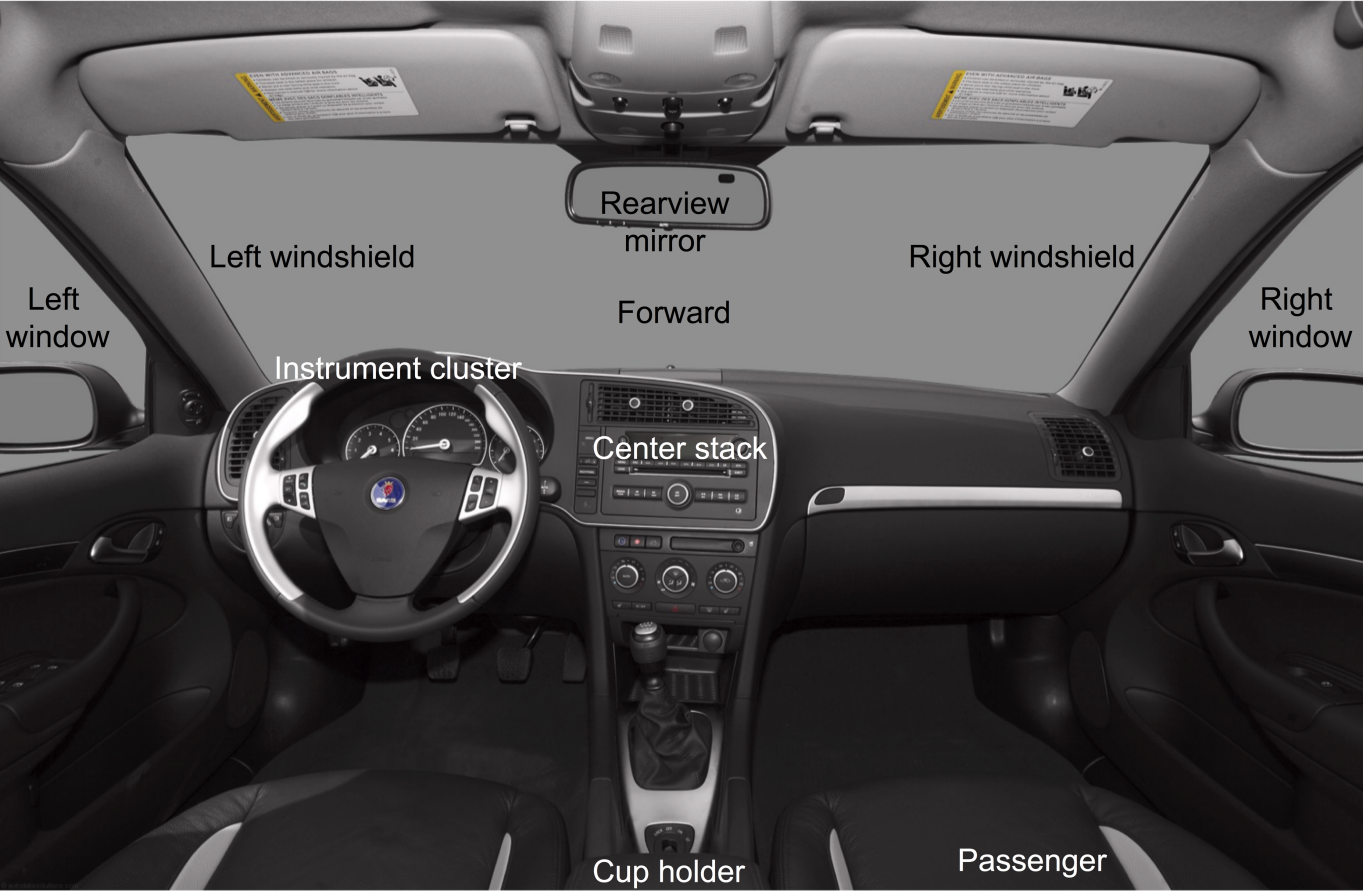
Glance locations for the manual coding.

Glance locations were coded by trained video analysts on a frame-by-frame basis into one of 16 locations: forward, left forward, right forward, rearview mirror, left window/mirror, right window/mirror, over-the-shoulder, instrument cluster, center stack, cell phone, interior object, passenger, no eyes visible—glance location unknown, no eyes visible—eyes are off-road, eyes closed, and other (e.g., any glance that cannot be categorized using the above codes). Figure 2 shows 10 of the 16 glance locations. A senior analyst reviewed the output of the coding and provided feedback to the less-experienced analyst. Glance allocations for each subject and task were merged with head rotation data using timestamps.

### Model training and validation

Training data were derived from the dataset by taking all data belonging to a randomly sampled subset of the subjects (80%). The data from the remaining subjects (20%) were used to build a validation dataset. As one of the tested classifiers (the Hidden Markov Model), takes the temporal structure of the input data into account, the timestamp ordering of the samples for each subject were maintained. All rotation variables were normalized by computing their individual *z*-scores per participant. The performance measures reported in the result section were computed using this normalization method. Furthermore, to discount any potential bias inherent in how subjects are sampled, a Monte-Carlo sampling technique was used (Muñoz et al., 2015; Muñoz et al., 2016). For each of 50 iterations of this sampling approach, training and validation test sets were generated as above. All models were trained, and performance values were then computed for each classifier as the mean of each performance metric (standard accuracy, F1 score, and Kappa value) over all iterations. One key issue considered is the unbalanced class structure (i.e., skewness) of the dataset, as glances to the forward roadway heavily outnumber glances to any other single location within the vehicle. For instance, out of all the glances to the forward roadway and center stack, approximately 95% belong to the former class (the forward roadway). Random subsampling was used to prune away the over-represented glance locations in the data.

### Data exploration

The intrinsic discriminative quality of the data plays a crucial role in any classification framework (i.e., classification may be difficult in datasets in which classes overlap strongly in feature space). Therefore, PCA (Jolliffe, 2005) was applied for representing the raw data in terms of its underlying structural patterns, computing the covariance matrix across all variables, and extracting the eigenvectors of this matrix. Given this information, which characterizes how statistical variance is distributed amongst (linear) combinations of variables, this analysis can identify and visualize properties that might have an impact on classification performance, in particular which variables are most likely to contribute to a high classification accuracy. As explained in ‘Principal component analysis’, in this context we use PCA to reduce the dimensionality of the data from three dimensions (rotations in *X*, *Y*, *Z* axes) to two dimensions and to examine which linear combinations of these rotations contribute most towards discriminating between glances to the center stack and forward roadway/right mirror.

### Model development

The classification methods presented in this paper are (a) k-Nearest Neighbor, (b) Random Forest, (c) Multilayer Perceptron, and (d) Hidden Markov Models. Implementations for all but the latter were taken from OpenCV*C* +  + library v.2.4.1, using default parameter settings unless otherwise noted. The HMM implementation was taken from MATLAB (2014). The parameters of each model were determined empirically with an experimental validation set (i.e., a random subset of the larger data pool). These methods were chosen based on the trade-off in running time, space complexity, and difficulty of parameter tuning. The k-Nearest Neighbor (kNN) algorithm has the lowest number of parameters (k, the number of neighbors to consider, and the distance metric to evaluate between points) and arguably the highest space and running time complexity requirements during evaluation, but is very fast during training. The Random Forest classifier (Breiman, 2001) is a representative ensemble method with high space complexity requirements both for training and evaluation, but unlike kNN it is both fast to train and fast to evaluate. Random forest uses a random subset of each input sample at different nodes to train sets of weak learners. This has the added benefit that as training progresses, variables with low information content are automatically filtered out, thus making the classifier especially well-suited for data structured across heterogeneous input variables. The main parameters that require tuning are the number trees in the forest, the depth of the trees (usually kept constant across all trees), and the function used to split nodes of each tree. The Multilayer Perceptron (MLP) was taken as a representative of the larger class of Artificial Neural Networks (ANN) for their ability to model non-linear relationships between data points. MLP space complexity is low for both training and evaluation, while running time is slow for training and fast for evaluation. The basic parameters that require tuning here are the number of hidden layers and the number of neurons in each layer. Hidden Markov Models (HMMs) (Rabiner & Juang, 1986) are employed to test how much of the classification signal lies in the temporal structure of the data. Sequences of head rotation and glance duration features are fed to the classifier, which then infers a single class label for the sequence of samples. Glance duration features are computed from glance allocations as the timestamp difference between adjacent glances in time and inform the classifier about how long each glance is (see Muñoz et al., 2016 for further details). As in the classical approach, one HMM is built from data from each class (glance location). The class label of an unobserved sequence is then determined by finding the HMM and its corresponding class that maximizes the log probability of the test sequence. Practically speaking, the only elemental parameter of an HMM is the number of hidden states used to model the temporal data. Muñoz et al. (2015) provides additional detail as to how the parameters for each model were set.

### Model performance measures

Generally, a sample corresponds to a single 3-tuple of *X*, *Y*, and *Z* rotations. This is the case for all classifiers except the HMM, which understands a sample as a sequence (in time) of these tuples. For the purpose of this study, these tuples we grouped according to subject, task, and glance location, ordered according to increasing time, and labeled with the label of the glance location used in the grouping. Classification then proceeded as in Muñoz et al. (2015). To assess performances of the classifiers, the following three performance measures were used in a binary classification framework (center stack vs. forward roadway, and center stack vs. right mirror). The reason for introducing multiple measures at this stage is to get a fair estimate of classifier performance in light of heavily skewed classes in the dataset (i.e., glances to the forward roadway have a much higher presence in the data than any other class):

1. Classification accuracy (AC) (e.g., Sokolova & Lapalme, 2009): the percentage of correctly classified samples (or sample sequences for the HMM classifier):
  \begin{eqnarray*}\text{Classification Accuracy}= \frac{\text{Number of correctly labeled samples}}{\text{Total number of samples}} \end{eqnarray*}
2. F1-score (FS) (e.g., Sokolova & Lapalme, 2009): a measure of how well the classifier was able to distinguish between classes given an unbalanced dataset.
  \begin{eqnarray*}\text{F1 score}& = \frac{2\times (\text{Positive predictive value}\times \text{Sensitivity})}{\text{Positive predictive value}+\text{Sensitivity}} \end{eqnarray*}
  \begin{eqnarray*}\text{Positive predictive value}& = \frac{\text{Number of true positives}}{\text{Number of true positives}+\text{Number of false positives}} \end{eqnarray*}
  \begin{eqnarray*}\text{Sensitivity}& = \frac{\text{Number of true positives}}{\text{Number of true positives}+\text{Number of false negatives}} \end{eqnarray*}
3. Cohen’s Kappa statistic (KP) (e.g., Carletta, 1996): a measure indicating how well a classifier agrees with a perfect predictor (higher values indicate high agreement).
  \begin{eqnarray*}\text{Cohen' s Kappa}= \frac{P \left( A \right) -P(E)}{1-P(E)} \end{eqnarray*}
  *P*(*A*) = Relative observed agreement between model and perfect predictor (i.e., accuracy)
  *P*(*E*) = Probablity of chance agreement between model and perfect predictor.

## Results

To answer the key questions outlined: (a) PCA was applied to the driving data (see ‘Test trials’ and ‘Model performance measures’) as a method of quantifying the contributions of each head angle (*X*, *Y* and *Z*) in their ability to discriminate between glance locations (this study looks at forward vs. center stack, center stack vs. right mirror), (b) several predictive models (‘Model development’) were tested (‘Model performance measures’) for predicting glance location based on head position while driving and their accuracies compared, and (c) individual differences in head-glance correspondence during driving were addressed.

### Principal component analysis

**Figure 3:**
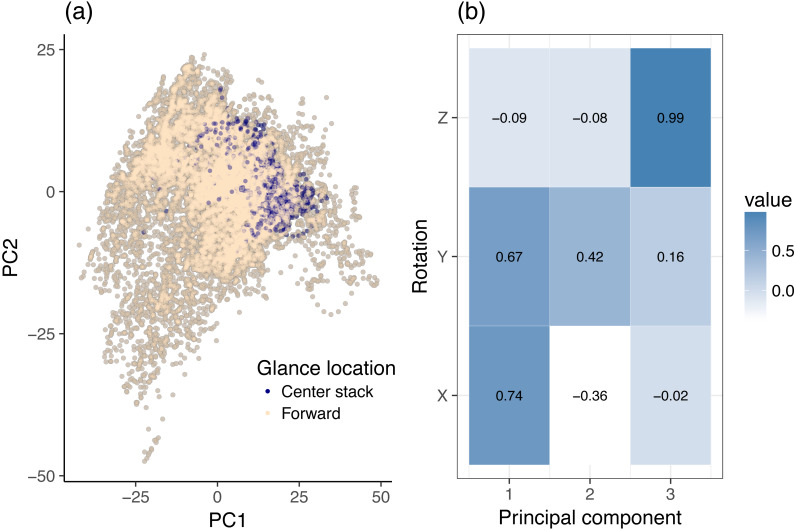
Principal component analysis (PCA) of dynamic data, using head rotation. (A) Glances to the center stack and forward roadway. (B) Principal components of head rotation *X*, *Y* and *Z* for all data samples belonging to the center stack and forward roadway class.

**Figure 4:**
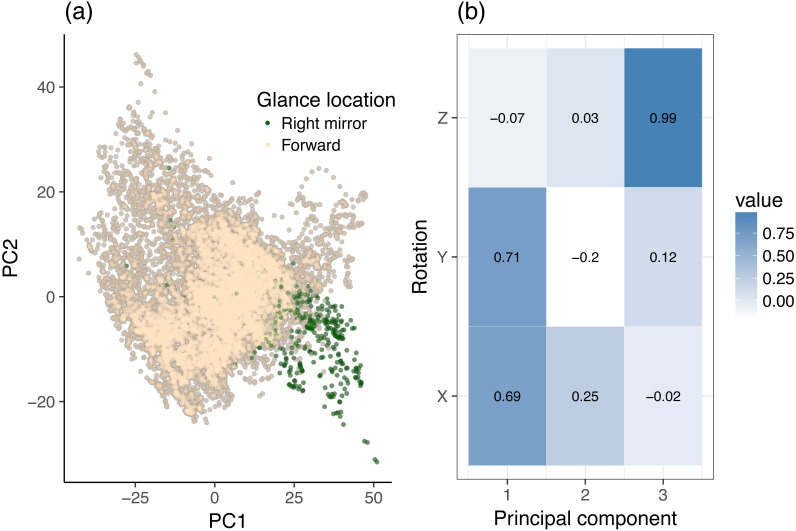
Principal component analysis (PCA) of dynamic data, using head rotation. (A) Glances to the right mirror and forward roadway. (B) Principal components of head rotation *X*, *Y*, and *Z* for all data samples belonging to the center stack and right mirror class.

The input to the PCA stage are the filtered *X*, *Y* and *Z* rotation variables. No additional signal filtering was applied beyond the original Butterworth filter used by the VTTI in the creation of the dataset. PCA was used to reinterpret the *X*, *Y* and *Z* filtered rotation variables along two independent (orthogonal) axes, i.e., the first two principal components. Figure 3A interprets the dynamic 2-class data (center stack vs. forward) as PCA scores. Each point on the graph corresponds to a single sample from the original dataset, irrespective of participant or task (but limited to glances to the center stack and forward roadway). Each axis of the graph corresponds to the noted principal component and illustrates the statistical behavior of the data along that component. Although the input data were standardized per participant, the values plotted have been de-normalized to aid interpretation: they therefore have magnitudes in the range of actual rotations. Figures 3A and 4A show the data along only the first two components of the PCA decomposition. The distribution of individual data points and their class correspondence in Fig. 3A was compared with the actual principal component values in Fig. 3B to establish an informal overview of which variables are most likely to contribute to the classification effort. A rough clustering of forward glances may be observed in Fig. 3A. This cluster center lies at moderate to high values of Principal Component 1 (PC1). Figure 3B reveals that PC1 increases in magnitude with increasing *X* and *Y* rotation, both of which load highly on this component. In this instance, a linear combination of *X* and *Y* rotation gives a rough but not clear cut decision boundary between glances to the center stack and glances to the forward roadway. PCA decomposition reveals that PC1, which may be interpreted as a linear mix of *X* and *Y* rotation, explains 66.5% of the variance of the dataset, while PC2 explains only 26.4% of the variance. Lastly, PC3 (which due to the high loading factor of *Z* rotation on this component may effectively be interpreted as the measure of the influence of *Z* rotation) contributes only 7.1% of the total variance.

The same analysis was made for the center stack vs. right mirror case. Figure 4 provides a two-dimensional sample distribution plot as well as the corresponding principal components. Figure 4A shows a nice separation of right mirror points, which are located towards on the bottom right quadrant of the scatter plot. Figure 4B reveals that *X* and *Y* rotation load highly on PC1, i.e., PC1 increases with increasing *X* and *Y* rotation, which makes sense intuitively. On the PC2 axis, the right mirror points are in negative PC2 space. Looking at Fig. 4B, we see that PC2 increases with increasing *Y* rotation and decreases with decreasing *X* rotation. Likewise, each component accounts for roughly the same amount of variance as in the center stack vs. forward roadway case above. We can therefore conclude that in order to distinguish glances to the forward roadway from glances the right mirror, it (a). Suffices to look at *X* and *Y* rotation, and (b). Right mirror points are highly correlated with high *Y* rotation (which loads heavily and positively on PC1 but negatively on PC2), but only for certain ranges of *X* rotation (which loads heavily and positively on both PC1 and PC2).

### Model validation

Table 1 presents the performance measures for all four classifiers using the two representations of the data (balanced vs. unbalanced) for the forward vs. center stack case. As noted earlier, Monte-Carlo sampling (50 iterations) was applied for deriving training and test sets. Using the balanced dataset that removed glance distribution bias during training leads to a higher performance in terms of sensitivity/specificity (F1 scores all ≥ 0.68) and prediction quality (Kappa all ≥ 0.41) of each classifier compared to (F1 scores all ≥ 0.04) and (Kappa all ≥ − 0.07) for the original unbalanced data. The HMM classifies sample sequences corresponding to blocks of data within a subject, task, and glance location group. Across all classifier, the relatively strong model performances may indicate that the temporal structure of head rotation features can be potential information source. Though all classifiers using the balanced dataset outperform a chance predictor, there is a clear upper bound on how much these features contribute to classification.

**Table 1:** Performance measures (AC, accuracy; FS, F1 score; KP, Kappa statistic) across all classifiers and class distributions for dynamic data, forward roadway vs. center stack.

	Original dataset			Balanced dataset		
	AC	FS	KP	AC	FS	KP
k-nearest neighbor	0.93	0.19	0.16	0.80	0.80	0.59
Random forest	0.86	0.30	0.24	0.79	0.78	0.59
Multilayer perceptron	0.78	0.29	0.22	0.80	0.82	0.59
Hidden Markov model	0.84	0.28	0.22	0.83	0.68	0.57

In addition, other locations within the vehicle were also tested against glances to the forward roadway in order to examine the relationship between classification accuracy and the visual angle of the target. HMM and Random Forest models, which showed relatively higher accuracy among other classifiers, were selected and tested. Table 2 places the previous center stack classification efforts in this context and gives performance measures for the two classifiers with the overall best performance. As expected, a rough correlation between increasing visual angle and classification accuracy may be observed, reaching up to 90% classification rate with a balanced dataset. The results may support that head pose data can detect particularly detrimental glances (i.e., high-eccentricity glances) with high accuracy, whereas using head pose data alone does not provide high accuracy to detect low-eccentricity glances.

**Table 2:** Performance measures (AC, accuracy; FS, F1 score; KP, Kappa statistic) across class distributions for the Random Forest and HMM classifiers for dynamic data, forward roadway vs. instrument cluster, vs. left mirror, vs. center stack, vs. right mirror.

Forward vs.	Model	Original dataset			Balanced Dataset		
		AC	FS	KP	AC	FS	KP
Instrument cluster	Random forest	0.59	0.33	0.08	0.56	0.48	0.11
Instrument cluster	Hidden Markov Model	0.66	0.32	0.12	0.66	0.61	0.33
Left mirror	Random forest	0.86	0.30	0.24	0.79	0.78	0.59
Left mirror	Hidden Markov Model	0.84	0.28	0.22	0.83	0.68	0.33
Center stack	Random forest	0.85	0.77	0.66	0.83	0.81	0.66
Center stack	Hidden Markov Model	0.83	0.72	0.60	0.85	0.83	0.69
Right mirror	Random forest	0.95	0.74	0.72	0.90	0.89	0.80
Right mirror	Hidden Markov Model	0.93	0.69	0.65	0.87	0.73	0.66

### Individual differences in head-glance correspondence

**Figure 5:**
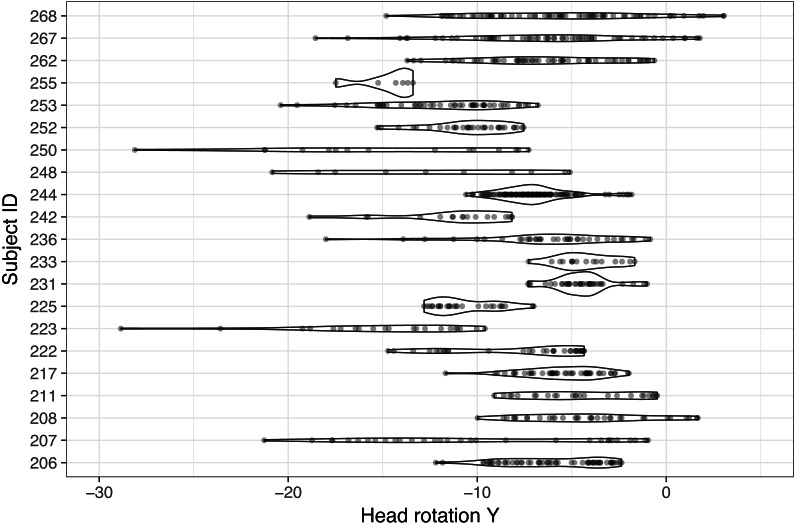
Comparison of individual distribution of *Y* rotation while glancing to the center stack during the radio task (note that one participant did not have any glances to the center stack, so this figure only shows 21 participants’ data).

Individual differences in head-glance correspondence were also tested. To minimize potential variability from characteristics of tasks, only the radio task (e.g., “Turn the radio on and then off”), which required glances to the center stack from the dynamic setting, was selected and analyzed. Figure 5 illustrates the distribution of 21 participants’ individual *Y* rotation while glancing to the center stack during the radio tasks (there was one subject who did not glance to the center stack and that case was excluded for the subsequent analysis). As can be observed in Fig. 5, a wide range of *Y* rotations exists while glancing to the center stack across the subject pool, with some subjects showing relatively narrow distributions and others showing wide distributions (note that individual dots in Fig. 5 visualize corresponding *Y* rotation values while glancing to the center stack). It is also important to observe that the center point of each subject’s distribution varies even they are looking at the same object in space.

**Figure 6:**
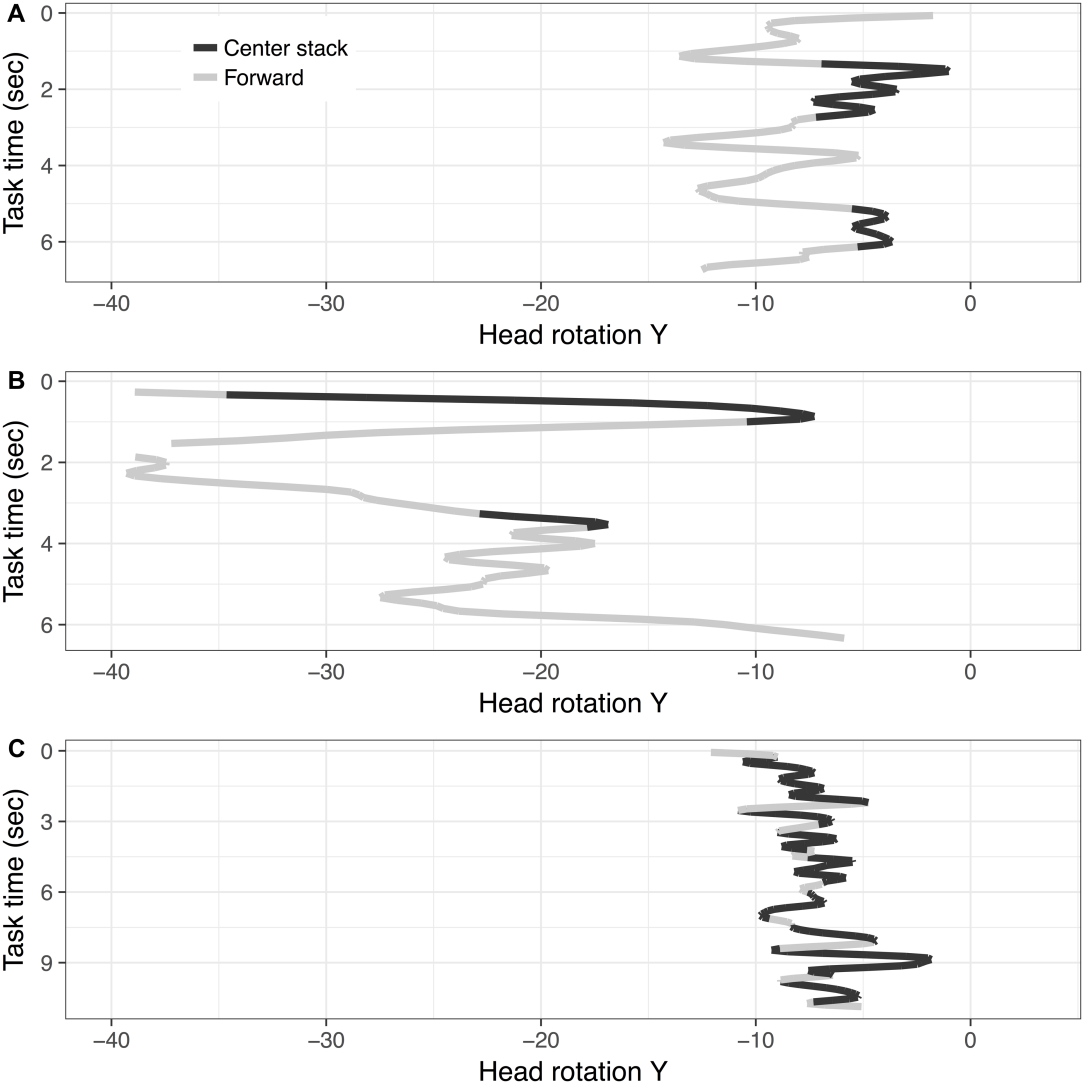
Illustration of three subjects’ *Y* rotation ((A) Subject 231, (B) Subject 250, and (C) Subject 244) over time during dynamic radio tasks (note: line color represents glance locations).

To further explore the differences in rotation distributions in glances to the center stack in relation to glances to the forward roadway, *Y* rotations were plotted over time while completing the radio task (see Fig. 6) for an illustrative sample of three subjects. This figure visualizes how drivers horizontally rotate (e.g., *Y* rotation) their head while engaging in the radio task and their glance locations over time (differentiated in colors). The top frame of Fig. 6 illustrates a profile that has relatively narrow range of *Y* rotation while glancing to the center stack, and (relatively) limited overlap between the ranges of *Y* rotation corresponding to glances to forward and glances to the center stack. The middle frame of Fig. 6 illustrates a profile that covers a wider range of *Y* rotation with significant overlap of the two glance locations. Finally, the lower frame illustrates a profile with a narrow range of *Y* rotation with a sizable overlap between the glance locations.

**Figure 7:**
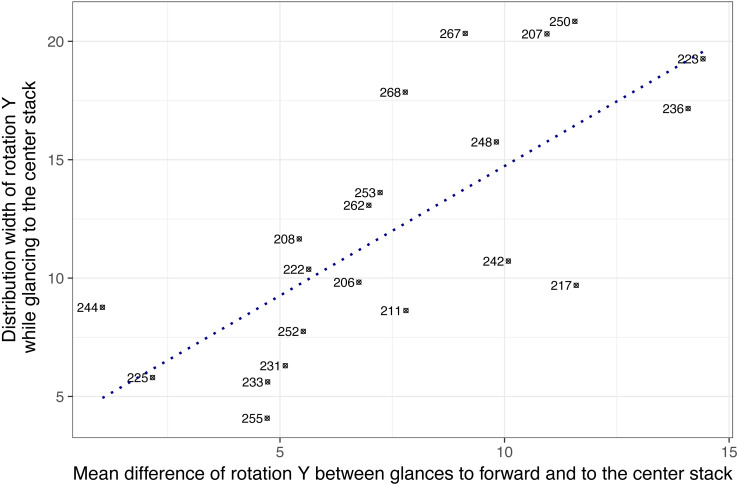
Drivers’ head angle profiles while glancing to the center stack during the radio tasks (note: numbers represent subject ID).

Based on these exploratory findings, it was assumed that individual difference in head-glance correspondence may exist. Figure 7 shows 21 subjects on two dimensions: (a) the mean difference of *Y* rotation between glances to forward and to the center stack, and (b) the range of *Y* rotation (i.e., distribution width of rotation Y while glancing to the center stack). The result showed that the two dimensions were positively correlated, *r*(19) = .73, *p* < .001, indicating that subjects who showed wider ranges of horizontal head rotations tended to have higher mean differences of rotation *Y* while glancing to forward and the center stack. For example, subjects 244 and 225 showed relatively narrow ranges of horizontal head rotations (less than 10 degrees) while glancing to the center and their mean rotation angles for glancing to the center stack were relatively close to their mean rotation angles for glancing to forward (the mean differences were 1.05 degrees for subject 244 and 2.16 degrees for subject 225). This may indicate that subjects on the left-bottom area in Fig. 7 such as subject 244 and 225 (i.e., narrow width and small mean difference) moved their head less actively to glance to the center stack, whereas subjects on the right-top are actively moved their head to glance to the center stack location.

## Discussion

This analysis investigated the relationship between head rotation and glance behavior during on-road driving. Various machine learning techniques were employed to examine the predictive value of head rotation for glance location at an individual level. In this particular example, we used PCA not specifically as a data reduction method (we only had three variables to begin with), but as a method to assess variable importance in the classification. We could clearly see how variances in *X* and *Y* rotation spread heavily across the first two components, i.e., in order to distinguish right mirror from forward glances it suffices to use a linear decision boundary on the first two components (this is evident from Fig. 3). That is, both vertical and horizontal head rotations are key variables to classify glance locations in this scenario, as expected. In a more general case, especially when the input data is highly dimensional, the outcome from PCA can inform what features to move forward with in any subsequent classification or regression task. For this particular scenario, it provides a step-up from working with the raw rotation values in two ways:

1. PCA provides an accurate 2D representation of this three dimensional problem, ironing out any inherent correlations between the *X*, *Y*, *Z* rotation variables in the process.
2. By demonstrating to what extent each rotation variable loads on each axis of this representation, and knowing beforehand how the samples in this representation map to glance locations (classes) in our classification framework, it can be easily shown which rotations or combinations thereof are likely to be of most help for a classifier trained to discriminate between these classes.

A total of four classifiers from a wide range of data interpretation techniques were used to detect patterns in head rotation data. Both unbalanced raw data, which included more cases of glancing forward than glancing to the center stack, and balanced data were tested. Substantial performance gains were observed when using the balanced training dataset. For the forward roadway vs. center stack case, Hidden Markov Models performed the best with an accuracy of 83%. When comparing this number to the remaining accuracy values it should be remembered that different ratios are expressed—HMMs work with sequences of point samples as inputs, while the remaining classifiers work with point samples directly. All of the modeling approaches provided results that were well in excess of chance findings, suggesting that head rotation data is a fairly robust predictive signal. Given that the limited number of glances to non-forward locations (i.e., glances to the center stack accounted for less than 5% of the total glances recorded) were captured during short/simple secondary tasks, model performance may be best considered as relative lower bound on the possible predictive quality for driver gaze detection. This study also looked at the variability in classification accuracy with increasing visual angles to show a significant correlation between the accuracy and visual angles.

There may be multiple factors that influence drivers’ head-glance correspondence such as: (a) road environment (e.g., highway driving vs. rural driving), (b) secondary-task characteristics (e.g., tasks require long off-road glances from drivers vs. tasks require short off-road glances), (c) individual strategies for interacting with secondary tasks (e.g., fixing a head to forward while glancing to the center stack), and (d) physical constraints. For this reason, we analyzed only one type of the secondary tasks (i.e., the radio task) for testing individual differences (note that only this analysis subsampled data for one task and other analyses used the entire dataset including all tasks). The result showed that individual differences in head-glance correspondence may exist. It is well known that owls have to turn their entire head to change views as their eyes are fixed in their sockets, whereas some lizards (such as Chameleons) have very large angles of eye movement. We also found lizard type drivers (e.g., subject 244 and 225 in Fig. 7) and owl type drivers (e.g., subject 223 and 236 in Fig. 7), and it was expected that head pose data could predict glance regions with higher accuracy for the owl type drivers who actively move heads while glancing away from the road. This result suggests the need for a user-specific model (e.g., training a classifier for each individual to detect glances away from the road by using head rotation) or additional input features (i.e., other facial features or pupil location) to increase model performance, especially for the lizard type drivers (who barely move their head while glancing away from the road). Also, predictive power of head rotation data for specific types of glances, such as longer off-road glances which have been linked to greater risk of collision (Victor et al., 2015), needs to be considered further.

Furthermore, efforts should assess the predictive power of head rotation data for certain types of glances such as those that are of longer duration, which have been linked to greater risk of collision (Victor et al., 2015).

One limitation of this work was that the analysis was only applied to the meidated and reduced data, given the fact that the present study conducted a secondary analysis of a subset of the original data. Therefore, it should be acknowledged that these findings might not be extrapolated to other circumstances such as a situation where estimation of head orientation is extremely challenging. Also, the present work focused on visual angle and classification accuracy between two objects, and individual differences in head and glance correspondence, regardless of task. However, as a previous study (Land & Tatler, 2001) revealed, drivers’ head movement and rotation pattern can be task-specific (e.g., racing). Therefore, further studies which expend to task characteristics (both primary and secondary tasks), will need to be undertaken for a deeper understanding of drivers’ head and glance correspondence.

## Conclusion

The present study investigated head pose data to test the feasibility of using head pose to predict glance location, and served as an exploratory base for developing approaches to building semi-automated glance annotation systems (Fridman et al., 2016a; Fridman et al., 2016b). This study also systematically tested factors that may affect model performance (e.g., data structure, visual angles between two glance locations, and individual differences in head-glance correspondence). This study achieved fairly accurate classification performance (e.g., classifying glances to forward vs. glances to the center stack), and supports the feasibility of detecting drivers’ glances away from the road by not using eye-tracking data. Especially, head pose data accurately classified glances to farther regions (i.e., high-eccentricity glances) from the center forward region. It can therefore be assumed that although classification accuracy for low-eccentricity glances is lower compared to the accuracy of high-eccentricity glances, the most detrimental glance regions (such as a center stack where the most current infotainment system are installed) can be detected by using head pose data. This work suggests that individual differences in head-glance correspondence may be separated into two classes and stimulated follow-on work that has been developed in Fridman et al. (2016b). However, from the data that is available, it is not clear if an individual can be “assigned” to one of the two classes (i.e., “owl” or “lizard”), or if there are more factors such as roadway conditions, secondary type interacting with some individual propensity for certain movement patterns.

This study used manually coded on-road data, which are relatively more valid and reliable than automatically tracked eye/head data from a driving simulator. Overall, this work suggests that head rotation data, a feature that may be recorded in the vehicle with limited sophistication using commercially available sensors, may provide a potentially lower cost and higher quality estimate of attention allocation than eye tracking data. Head movements may be used to fairly reliably predict safety critical off-road glances to regions in the vehicle frequently associated with in-vehicle distractions.

##  Supplemental Information

10.7717/peerj-cs.146/supp-1 Supplemental Information 1 Head pose estimation validation data This data set includes separate files for eye glance, facial annotation, head rotation, etc. Background and detailed information of each variable are described in the data dictionary file.

## Additional Information and Declarations

### Data Availability

The following information was supplied regarding data availability: The raw data has been supplied as a Supplemental File.
